# Insights on the Interplay between Cells Metabolism and Signaling: A Therapeutic Perspective in Pediatric Acute Leukemias

**DOI:** 10.3390/ijms21176251

**Published:** 2020-08-28

**Authors:** Laura Anselmi, Salvatore Nicola Bertuccio, Annalisa Lonetti, Arcangelo Prete, Riccardo Masetti, Andrea Pession

**Affiliations:** 1 Pediatric Hematology and Oncology Unit, S.Orsola-Malpighi Hospital, University of Bologna, 40126 Bologna, Italy; laura.anselmi@aosp.bo.it; 2 Pediatric Hematology-Oncology Unit, Department of Medical and Surgical Sciences DIMEC, University of Bologna, 40126 Bologna, Italy; arcangelo.prete@aosp.bo.it (A.P.); riccardo.masetti5@unibo.it (R.M.); andrea.pession@unibo.it (A.P.); 3 Giorgio Prodi Interdepartmental Cancer Research Centre, University of Bologna, 40126 Bologna, Italy; annalisa.lonetti2@unibo.it

**Keywords:** metabolism/metabolomics, leukemia, pediatric tumors, molecular approaches, signal transduction inhibitors

## Abstract

Nowadays, thanks to extensive studies and progress in precision medicine, pediatric leukemia has reached an extremely high overall survival rate. Nonetheless, a fraction of relapses and refractory cases is still present, which are frequently correlated with poor prognosis. Although several molecular features of these diseases are known, still the field of energy metabolism, which is widely studied in adult, has not been frequently explored in childhood leukemias. Metabolic reprogramming is a hallmark of cancer and is deeply connected with other genetic and signaling aberrations generally known to be key features of both acute lymphoblastic leukemia (ALL) and acute myeloid leukemia (AML). This review aims to clear the current knowledge on metabolic rewiring in pediatric ALL and AML, also highlighting the influence of the main signaling pathways and suggesting potential ideas to further exploit this field to discover new prognostic biomarkers and, above all, beneficial therapeutic options.

## 1. Introduction

Leukemia is the most common cancer in children and teens (20–30% of pediatric cancers). Within these, acute lymphoblastic leukemia (ALL) is the most represented, mainly in children ranging from 2 to 5 years of age, while most of the other cases are acute myeloid leukemia (AML), largely present within the first 2 years of age and in teenage years. Even though improvements in treating these cases have been achieved, especially through multiagent chemotherapy, with an overall survival rate of 90% for ALL patients and 75% for AML patients, still a consistent fraction of refractory disease or relapse remains [1,2,3,4,5]. A comprehensive knowledge of leukemia molecular biology is fundamental, on the one side to predict treatment effectiveness, and on the other side to identify novel therapeutic targets that will help to overcome drug resistance but also limit side toxicity [6,7].

In this context, cellular metabolism has gained a large amount of attention throughout the years, even though still very little is known about its role in pediatric acute leukemias.

Cells transformation during tumorigenesis involves a massive number of alterations, and the connection between cancer cells and modification of cell metabolism is well known. For almost 90 years since Otto Warburg defined the so-called “Warburg effect”, metabolic rewiring has been widely studied and described in multiple cancer models [8].

Cancer cells need a high amount of energy to boost their rapid growth and proliferation, and they also need to escape normal cell cycle checkpoint controls that normally would regulate these phases only in stressful metabolic conditions. Their metabolic phenotype is controlled by both intrinsic genetic mutations and external responses to the microenvironment [9].

Increased glucose uptake and aerobic glycolysis have been confirmed to be a hallmark of leukemic cells [10]; however, more recent findings revealed that functional mitochondria and oxidative phosphorylation (OXPHOS) are fundamental as well [11]. Different non glycolysis-derived mechanisms can provide intermediates to sustain the tricarboxylic acid (TCA) cycle, such as fatty acid oxidation, amino acid oxidation and glutamine [12].

Moreover, different signaling pathways contribute to altering the metabolic homeostasis, and most of them are frequently dysregulated in the context of pediatric acute leukemias, such as the PI3K/Akt/mTOR pathway, MYC, FLT3 and RAS [13].

**Figure 1:**
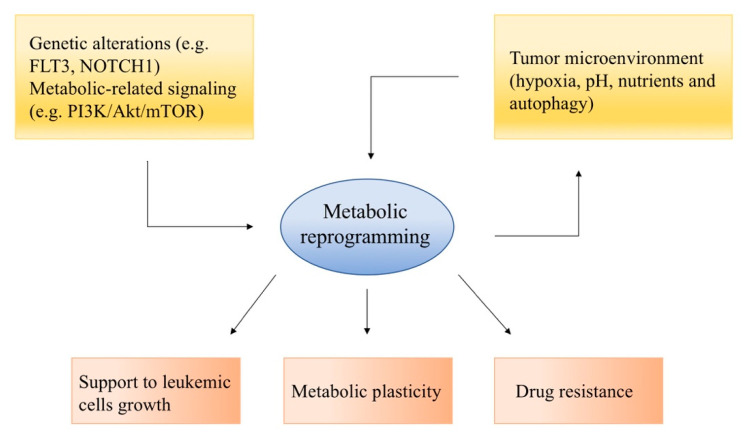
Metabolic rewiring in cancer cells is driven by multiple factors, involving both cytogenetic alterations, aberrant signaling and tumor microenvironment, and this eventually leads to molecular mechanisms that support cancer cell proliferation and therapy resistance.

Knowing and understanding these intricate networks (Figure 1) represent a powerful opportunity to find novel therapeutic targets and an additional tool to discern the mechanisms responsible for drug resistance, leukemic cell survival and plasticity.

## 2. Metabolic Reprogramming in Acute Leukemias

The normal regulation of hematopoietic stem cells (HSC) metabolism involves an overall upregulation of glycolysis over OXPHOS, due to their need to proliferate and differentiate in a hypoxic niche [14]. Stimulation of glycolysis is driven by hypoxia inducible factors (HIF-1s), while another important process is represented by fatty acid oxidation (FAO), which also regulates the balance between stemness and commitment of HSC, and is positively regulated by an energy sensor, LKB1 and its downstream target AMP-activated protein kinase (AMPK), which is a master regulator of catabolic processes [15].

As previously cited, the aerobic glycolytic rate, or Warburg effect, is increased in most AML and ALL cells. In particular, leukemia-initiating cells (LIC), which are a rare subpopulation of leukemic cells characterized by leukemia initiation properties, stemness and drug resistance [16], rely on a high glycolytic rate [17], which was demonstrated to be a central mechanism in AML leukemogenesis driven by the fusion proteins BCR-ABL and MLL-AF9 oncogenes [18]. These proteins are the results of genomic translocations, hence BCR-ABL originates from t (9;22) and the so-called Philadelphia chromosome, which is mostly found in chronic myeloid leukemia but occasionally also in AML and ALL cases, while MLL-AF9 derives from t(9;11) and is the most frequent mixed-lineage leukemia (MLL) gene rearrangement in childhood AML [19,20,21]. Glucose transporter genes, as well as other components of the glycolytic cascade, are upregulated. Lactate production from glucose occurs 10 up to 100 times faster than its complete oxidation in the mitochondria, and this can confer a selective advantage to cancer cells. In this scenario, the increased glucose consumption is used as a carbon source for anabolic processes needed to support uncontrolled proliferation. Mammalian target of rapamycin complex 1 (mTORC1) is a nutrient sensor activated downstream of the PI3K/Akt signaling pathway; it promotes protein and lipid biosynthesis, stimulates mRNA translation and indirectly promotes hypoxia-inducible factor 1 (HIF1) activity, whose related metabolic changes are a major determinant of the glycolytic phenotype [22]. PI3K cascade, frequently overexpressed in leukemia, contributes to altering the metabolic homeostasis also through Akt1 activity, which stimulates glycolysis by increasing the expression and membrane translocation of glucose transporters, and by phosphorylating key enzymes such as hexokinase and phosphofructokinase 2 [23].

Another relevant energy sensor is the AMP-activated protein kinase (AMPK), which acts as a counterpart of Akt- and mTOR-mediated effects in response to energetic stress, and specifically to a high AMP/ATP ratio, by shifting towards a OXPHOS phenotype and inhibiting proliferation [15]. For its activation, AMPK needs the phosphorylation of liver kinase B1 (LKB1), and the related gene is often mutated, suppressing AMPK effects.

In roughly 60% of T-ALL cases, there is an aberrant expression of NOTCH1, a member of the Notch family of type I transmembrane proteins that function as receptors for ligands expressed neighboring cell surfaces (named Delta-like and Jagged in mammals) and whose signaling plays a fundamental role in cells by influencing the self-organization of cell diversity to the differentiation and proliferation, their behavior and morphology. NOTCH1 promotes glycolysis, mostly mediated by Myc, but also by AMPK activation, that through inhibition of mTORC1 favors oxidative metabolism and mitochondrial complex 1 activity over aerobic glycolysis, which is shown to be restrained compared to glycolytic rate in normal proliferating T cells [24]. AMPK seems to have a dual role, as on one side, it seems to suppress cancer cell proliferation by leading to mTORC1 inhibition, p53 activation and other transcriptional responses, while on the other side, it helps to promote cancer cell survival by activating ATP-generating pathways (such as glycolysis, fatty acid uptake and oxidation and mitochondrial biogenesis) [15].

Nonetheless, the role of mitochondria remains relevant, and specific subgroups of leukemic cells in the leukemic niche show different metabolic dependencies, such as leukemic stem cells (LSCs, also referred to as the leukemia-initiating cells LICs), which exhibit higher levels of Bcl2-mediated OXPHOS [25]. AML cells resistant to cytarabine show remarkable OXPHOS activity, and targeting their mitochondria sensitizes them to chemotherapy [26].

Another key metabolite for both AML and ALL cell growth is glutamine [27]. Glutamine is the most abundant circulating amino acid in blood and muscle and is metabolized within the mitochondria through an enzymatic process termed glutaminolysis. Indeed, glutamine, once entered inside the cells through specific transporters such as ASCT2 and SN2, is converted to glutamate by the glutaminases. Glutamate, by being transformed to α-ketoglutarate thanks to either GLUD or transaminases, can also feed the TCA cycle through a process called anaplerosis, which actually permits, under limited pyruvate availability due to aerobic glycolysis, the maintenance of mitochondrial homeostasis and functionality [28].

Although metabolism has been widely studied in adult leukemias, to date, a limited number of studies can be found about the abovementioned mechanisms in the context of pediatric acute leukemia cases. With this review, we aim to clear the current available literature on this subject, focusing on ALL and AML.

## 3. Current Knowledge on Pediatric Acute Leukemia

### 3.1. Metabolic Hallmarks of Pediatric ALL

The complexity of deregulated metabolic networks, previously described, is a hallmark of ALL cells as well. Unveiling the reprogramming of cellular energetic homeostasis in leukemic cells might contribute to identifying novel therapeutic targets. Although some recurrent aberrations, directly or indirectly linked to metabolic changes, have been identified and already targeted through therapeutic strategies, still around 20% of ALL pediatric cases relapse and undergo drug resistance [29]. Likely, this is at least partially caused by metabolic plasticity, through which leukemic cells achieve fuel productions by switching and alternate different energy sources. A more comprehensive understanding of metabolism and metabolic-related signaling will contribute to recognizing the machinery involved in refractory disease and relapse, thus suggesting novel molecular targets.

In a comprehensive study conducted on pre B-ALL patient cells compared to healthy CD34^+^ bone marrow cells, leukemic cells displayed a higher glycolytic phenotype. These cells express higher levels of glucose transport genes GLUT1 and GLUT4, and phosphofructokinase PFKL, while components of the TCA cycle (such as the lactate dehydrogenase LDHB) are downregulated. At the same time, lactate production rate is extremely high, even in normoxic conditions, overall suggesting a reduction in oxidative phosphorylation (OXPHOS) [30]. Accordingly, pathogenic mutations in mitochondrial DNA (mtDNA), which encodes for 13 essential subunits of the OXPHOS machinery, have been described analyzing a panel of 36 childhood ALL samples. In 8 out of 36 patients, missense or nonsense mutations in mtDNA (components of ETC-Complex I, Complex IV and the ATP synthase) were found, and 6 out these 8 patients died from ALL. Within the whole group, there was a correlation between a poor prognosis at diagnosis due to genetic markers and a mutated mtDNA pattern [31].

#### 3.1.1. Treatment Efficacy Prediction

A deeper understanding of metabolic networks in ALL is also important to predict prognosis and treatment efficacy. However, most of the metabolic dysfunctions are caused by aberrant expression of genes and/or proteins not directly involved in cell metabolism. In 40%–50% of patients with childhood ALL, mutations in RAS pathway, involved in cell growth, survival and differentiation, can be found, and these are correlated with a poor prognosis [32,33]. Intriguingly, marked changes in metabolic homeostasis are found in RAS-mutated ALL patients [34], with a general switch to a hyperglycolytic phenotype accompanied by a decrease in mitochondrial OXPHOS rate [35]. The use of glycolytic inhibitors, such 2-deoxy-d-glucose (2-DG) which showed very low levels of side toxicity, could represent an interesting therapeutic approach [36]. A comprehensive study based on a panel of 187 children with pre-B ALL showed—through massive RNA-seq, ChIP-seq and metabolic analysis—that a signature of mutations or deletions in four specific transcription factors correlates with glucose metabolism reprogramming and, thereby, with a poor response to glucocorticoids (GC) treatment [37]. GCs such as prednisolone and dexamethasone are involved in standard therapeutic protocols, which are extremely effective, and response and sensitivity to GC treatment are positive predictors of patient outcome [1]. However, very little is known about GC mechanisms involved in triggering leukemic cells apoptosis, and most importantly, those mechanisms that might be involved in GC resistance, as around 20% of ALL pediatric cases relapse and often suffer from treatment side effects [38]. A recent study finely described the metabolic reprogramming taking place in leukemic cells in response to GC treatment that favors a switch from anabolism to catabolism, which ultimately leads to an autophagy-mediated cell death. This switch starts with a MYC-mediated downregulation of glucose uptake, followed by an upregulation of enzymes involved in fatty acid oxidation and glutamine synthesis, which seems to be responsible for the modulation of the autophagy processes [39]. Low expression of the glutamine-synthesizing enzyme glutamate-ammonia ligase is predictive of poor outcome in ALL [40]. Taken together, these results clearly emphasize the relevance of many genetic metabolic-related aspects when a specific therapy regimen needs to be considered.

#### 3.1.2. Metabolic Inhibitors

Extensive studies have highlighted the efficacy of different kind of inhibitors affecting energy metabolism. 2-DG, an inhibitor of hexokinase and thus of the glycolytic flux, induces apoptosis in combination with the GC methylprednisolone, and Leni et al. showed that this apoptotic effect is mainly due to 2-DG inhibition on N-linked glycosylation rather than its inhibition of glycolysis [41]. In this way, 2-DG is able to sensitize to GC treatment resistant childhood ALL cell lines. 2-DG interferes with N-linked glycosylation by competing with the endogenous substrate mannose with its structural similarity, even though this leads to a downregulation of the production of all glycosaminoglycans, proteoglycans and glycolipids, and thus to the blocking of protein synthesis enhanced by the promotion of endoplasmic reticulum (ER) stress and the subsequent activation of unfolded protein response (UPR)-mediated apoptosis [41]. Those mechanisms are of particular interest because by helping the cell to respond to a metabolic stress, they promote leukemic cell survival. Potential therapeutic strategies also involve mitochondria. As a matter of fact, even though the occurring metabolic rewiring is frequently related to glycolysis and glutaminolysis upregulation, functional mitochondria are still present in leukemic cells, and the electron transport chain can be targeted. The use of metformin, which inhibits complex I, exhibited a cytotoxic effect in a mouse model of *PTEN*-deleted T-ALL, and it synergized with 2-DG [42]. It also showed a synergistic effect in combination with anthracyclins and daunorubicin in primary blasts from childhood ALL patients [43,44]. More interestingly, very promising results were obtained from a Phase I trial involving metformin in combination with standard chemotherapy in a group of 14 relapsed or refractory ALL pediatric patients [45].

It is important to mention another standard treatment of pediatric ALL which is directly linked to cellular metabolism of leukemic cell, L-asparaginase (ASNase) [46]. It catalyzes the hydrolyzation of asparagine and glutamine, and its therapeutic rationale was historically based on the fact that cancer cells express low activity of asparagine synthetase (ASNS) [47]. ASNS is an enzyme that catalyzes the ATP-dependent biosynthesis of L-asparagine from L-aspartate, an amino acid required in a large amount in cancer cells, probably for its role in maintaining cellular amino acid homeostasis, and cells lacking the enzyme depend on extracellular asparagine supply. The correlation between ASNS expression and the sensitivity to L-Asparaginase treatment is controversial. Indeed, it has been reported that asparagine synthetase levels do not correlate with resistance to ASNase [48]. On the other hand, it has been demonstrated that the methylation status of ASNS correlated with the low expression of the gene and the sensitivity to L-Asparaginase [29]. In turn, ASNase treatment induces fatty acid oxidation accompanied by glycolysis and lactate production reduction, with an overall increase in oxygen consumption, not present in normal B lymphocytes. The effect on protein translation, de novo pyrimidine synthesis and fatty acid oxidation is mediated by mTORC1 and a related GTPase RagB, while the inhibition of glucose uptake is mediated by the downregulation of Myc. Interestingly, pharmacological inhibition of fatty acid oxidation through etomoxir used in combination with ASNase synergistically induced leukemic cells death, verified in a panel of BCP-ALL patient samples [49]. The latter study represents an extraordinary example of how important it is to investigate the metabolic reprogramming occurring in response to standard treatments, which might explain rescue pathways adopted by cells that might eventually overcome therapy resistance, and above all, might indicate promising new therapeutic approaches, such as the use of fatty acid inhibitors together with ASNase [49]. Nevertheless, it is also critical to integrate data from epigenetic studies, as the control of gene expression is directly or indirectly linked to leukemogenesis and metabolic response, as demonstrated by the use of the histone deacetylase (HDAC) inhibitor Vorinostat. This drug is able to reexpress a number of deregulated genes involved in GC response, nucleotide biosynthesis and folate metabolism, and specifically these metabolic genes resensitize cell response to Prednisolone [33,50,51].

#### 3.1.3. Metabolic-Related Signaling Pathways

An important role in contributing to metabolic rewiring is also played by multiple signaling pathways, aberrantly overactivated in ALL cells and involved in cellular metabolism. Thanks to genomic and biochemical assays, the PI3K/Akt/mTOR pathway has been found to be upregulated both in pediatric B and T-ALL. Phosphatidyl-inositol-3-kinase (PI3K) is recruited to the membrane upon activation of growth factor receptor protein tyrosine kinases (RTKs), and it leads to the production of phosphatidylinositol-3,4,5-triphosphate (PIP_3_) which in turn recruits proteins with the pleckstrin homology (PH) domain, such as phosphoinositide-dependent kinase 1 (PDK1) and Akt/protein kinase B (PKB). Akt, to be fully active, needs to be phosphorylated by both PDK1 and mTORC2 on two different residues, thus enabling its multiple targets phosphorylation and subsequent downstream regulation of proliferation, metabolism and many vital cellular functions. The core component of the mTORC2 complex is the mammalian target of rapamycin (mTOR), which is also present in mTORC1, a nutrient/energy/redox sensor which controls protein synthesis (Table 1) [52]. Akt stimulates glycolysis by increasing the expression and membrane translocation of glucose transporters, and by phosphorylating key enzymes such as hexokinase and phosphofructokinase 2 [53]. Activated mTOR further promotes protein and lipid biosynthesis; it stimulates mRNA translation and indirectly promotes hypoxia-inducible factor 1 (HIF1) activity, whose related metabolic changes are a major determinant of the glycolytic phenotype [54]. In specific subgroups such as Philadelphia chromosome-positive (Ph^+^) ALL, the BCR-ABL1 fusion protein activates the PI3K/mTOR pathway [55]. The efficacy of mTOR inhibitor sirolimus (one of the so-called “rapalogs”) in abrogating leukemic cells expansion has been tested in vivo in pediatric Ph-like ALL xenografts, a recently described subtype of pediatric high-risk B-precursor ALL (B-ALL) which exhibits a gene expression signature similar to Ph^+^ ALL with a poor prognosis, even though the therapeutic potential of this approach remains preclinical [56].

In T-ALL, one of the most frequently mutated genes, which strongly affects the metabolic flux as well, is *NOTCH1*. NOTCH1 binds to specific receptors, and it is internalized after cleavage by gamma secretase. An exhaustive metabolomic research project, focusing on gamma secretase inhibitor (GSI) resistance in a T-ALL mouse model, demonstrated that in *NOTCH1*-driven T-ALL, the mutational background of *PTEN*, a negative regulator of the PI3K/Akt cascade, contributes to a metabolic rewiring as a consequence of GSI treatment. A lack of PTEN induces increased lactate levels and increased anabolic processes (such as ribosomal RNA processing, amino acid and nucleotides synthesis) [59]. A key role in *NOTCH1*-induced T-ALL is also played by glutamine, which through glutaminolysis is one of the main carbon sources, particularly by feeding the TCA cycle, while glucose is mostly converted into lactate in these cells [13]. The use of BPTES, a potent glutaminase inhibitor, has synergistic effects with GSI therapy, even though in *PTEN*-null cells the effect is abrogated, consistent with the fact that PTEN is a key player in regulating downstream a hyperglycolytic phenotype that compensate glutamine absence to feed metabolic processes (Table 1) [59].

Moreover, an analysis of 44 pediatric T-ALL samples showed that 21 out of 44 patients displayed mutations along the PI3K/Akt signaling pathway, and those with complete loss of *PTEN* correlated with a poor clinical outcome (chemotherapy induction failure) [65].

A comprehensive summary of different metabolic-based therapeutic approaches that could be further studied and exploited in pediatric leukemia are resumed in Table 1.

### 3.2. Metabolic Hallmarks of Pediatric AML

#### 3.2.1. From Cytogenetic Aberrations to Metabolic Response

Only in recent years researchers have started to focus on metabolic studies also in pediatric AML. Some of the most frequent aberrations occurring—such as FMS-like tyrosine kinase 3 (FLT3), a major driver of oncogenic signaling in AML and one of the main prognostic markers—finely regulate cell proliferation, differentiation and survival, thus indirectly acting also on the modulation of energy metabolism [66]. However, metabolomic studies have been reported mostly in adult AML cases, while evidence in pediatric AML is poorly present.

Recently, a metabolomic study analyzed a cohort of 16 pediatric AML patients, evenly distributed in wild-type FLT3 and mutated FLT3-ITD, and asserted a direct correlation between metabolic features and FLT3 mutational background, with 209 features that were differentially expressed between the groups. Samples with FLT3-ITD displayed a deregulation in metabolic pathways involving cysteine and methionine, purine metabolism and biosynthesis, fatty acid metabolism and several other amino acids metabolic pathways as well as intermediates of glycolysis and TCA cycles. In particular, purine, cysteine and methionine metabolism turned out to be significantly influenced by FLT3-ITD mutation [67]. It is not surprising that an accumulation of nucleosides occurs as probably a direct readout of a more prominent proliferation rate in these cells compared to their wild-type counterpart. Interestingly, some of the metabolites involved, such as tryptophan, succinate or 2-hydroxyglutarate, are key features of metabolic rewiring, which in turn is a crucial aspect of leukemogenesis. L-carnitine, responsible for fatty acid transportation across the mitochondrial membrane, was considerably higher in FLT3-ITD, a result which is in line with other reports on different types of cancer and that indicates carnitine as a potential useful biomarker in pediatric AML with FLT3-ITD [67,68].

Some of the cytogenetic aberrations in pediatric AML include alterations in genes involved in energy metabolism. This is the case of the ASNS gene, which is down regulated when monosomy of chromosome 7 occurs, as this and other genes are located in the deleted region. This aberration is frequent in high-risk de novo AML and therapy-related myeloid neoplasm patients. In this context, the use of ASNase, which hydrolyzes L-asparagine to L-aspartic acid and ammonia, hence depletes L-asparagine pool and thereby induces tumor cell death. In a fine study, after demonstrating that ASNS haploinsufficiency is directly caused by chromosome 7 deletion in cases of monosomy, the ASNS gene was silenced in a panel of AML cell lines by means of RNA interference techniques. Afterwards, cells were treated with L-Asp for 48 h showing significant cytotoxic effects compared to control [58]. This study was the first to highlight a potential use of L-Asp as a chemotherapeutic agent not only in the context of ALL but also in AML, in particular in a poor prognosis karyotypic subtype. When conventional chemotherapy is not an efficient option, the necessity of novel biomarkers and subsequently of novel drugs that might overcome the poor outcome emerges. In this context, the bone marrow microenvironment seems to play a determinant role. A very recent research project suggests that protective signals within the stromal microenvironment could maintain residual leukemic cells relatively insensitive to ASNase therapy and be potentially responsible for the recurrence of the disease. Indeed, mesenchymal stromal cells derived from healthy donors or from AML patients displayed a high expression of ASNS [69]. Moreover, it has been demonstrated that the monocytic cells resident in the bone marrow can produce *CTSB*, a lysosomal cysteine protease. The CTSB mediated the degradation L-Asparaginase and mediated the resistance to drug treatment as well. The treatment with CTSB-specific inhibitor can partially reestablish the sensitivity to L-Asparaginase treatment [70].

Targeting the deregulated metabolism with specific inhibitors also represents a promising tool for myeloid leukemias, and several of the abovementioned drugs used in ALL can be exploited in AML as well. For instance, 2-DG, through N-linked glycosylation inhibition, is able to abrogate cell surface expression and signaling of FLT3-ITD and mutated c-kit in a panel of AML cell lines and primary blasts from patients, thus inducing cell death [57]. This option opens an intriguing window on new therapeutic options, in particular, in patients with mutated receptor tyrosine kinases (RTKs) and those resistant to some tyrosine kinase inhibitors, such as quizartinib. Nonetheless, it is important to mention another compound that was not previously cited, IACS-010759. This drug is a mitochondrial complex I inhibitor and it showed strong cytotoxic effects in models of AML reliant on OXPHOS, and it is currently under evaluation in a Phase I trial in relapsed/refractory AML [60] (Table 1).

#### 3.2.2. Metabolic-Related Signaling Pathways

If oncogenic signaling plays a key role in orchestrating T-ALL cell survival, proliferation and metabolism, this is equally true for AML as well. As previously seen, FLT3 represents an example of how a signaling network can contribute to metabolic reprogramming, which in turn favors therapy resistance/sensitivity. Furthermore, the PI3K/Akt/mTOR signaling pathway is also aberrantly activated in a large number of AML patients, mostly due to alterations in growth factors and mutations in FLT3 itself, c-kit and Ras [69]. When Akt is activated, mTORC1 increased signaling promotes HIF-1 expression through 4EBP1 and eIF4E, and glucose transporter 1, thus enhancing glycolysis [71]. Constitutive activation of mTORC1 has been found in AML cells, which contributes to their glucose dependence but also to an altered protein synthesis that favors leukemic cells growth [72]. Additionally, in this context, the bone marrow microenvironment could play a determinant role. Indeed, the interaction with bone marrow with leukemia cells mediates the activation of the AKT pathway as well as the resistance to cytarabine treatment [73].

Different inhibitors of the Akt/mTOR axis have been studied in AML and demonstrated to have cytotoxic activity [74]. Inhibitors of the mTORC1 complex, the previously cited rapalogs, showed promising results, evidenced in a study conducted by treating AML primary blasts with Everolimus [75]. Nonetheless, to date, these drugs used in combination with chemotherapy in clinical trials for refractory/relapsed patients have not confirmed the expected preclinical results [61]. Other inhibitors that have been extensively studied in vitro to be more efficient are dual PI3K/mTOR inhibitors, which prevent any negative feedback mechanism that might reactivate Akt activity. These include NVP-BEZ235, BGT226 and PI-103, tested in AML cell lines and primary blasts from patients with promising results [62,63,64,76]. What emerged from different research, in line with what was evidenced in ALL, was that as a consequence of the treatment, autophagy mechanisms developed. Autophagy is an important cellular response to metabolic perturbation which eventually can prevent leukemic cell death by helping cells to adapt to metabolic stress and drug exposure [77]. Preliminary studies that combined signaling inhibitors with autophagy inhibitors, such as chloroquine, induced a more adequate effect in inducing AML apoptosis [78,79].

## 4. Conclusions

In this review, we aimed to share the current, although not so vast, literature on a deeper knowledge of cellular metabolism and how the main signaling pathways interact with the metabolic network. This is of fundamental importance to find novel therapeutic approaches in pediatric acute leukemias and also to better understand treatment resistance mechanisms, which represent the main issue in pediatric hematological diseases.

If oncogenic abnormalities have already proved their strong rationale for developing specific inhibitor-based therapies, still other interrelated aspects, such as metabolic implications, need to be considered and might be useful to set up proper personalized combination approaches. These options could synergize with standard chemotherapy regimens or provide an alternative to be able to optimize therapies for responsive patients by diminishing side toxicities, but also to avoid relapses and refractory diseases [72].

In summary, if we could give an idea of future perspectives on the subject, further improvements in metabolic studies should be performed in the pediatric leukemia setting. An ever increasing amount of research in adult AML and ALL has been conducted in recent years, which has suggested some interesting inhibitors (e.g., OXPHOS, glutaminase or glycolysis inhibitors) that could synergize with standard chemotherapy and reduce side effects by enhancing specific targeting. Even if the pediatric background is different, in particular regarding the overall survival rate and the efficacy of current treatment protocols, still the analysis of cell metabolic rewiring (e.g., through metabolomics studies in mass spectroscopy, L-Lactate assays, use of a Seahorse XF Analyzer), together with related signaling pathways (e.g., phosphoproteomic studies), can be extremely useful to understand general response mechanisms to treatments in leukemic cells, to find novel predictive biomarkers, and thus, to enlarge our general knowledge about these pathologies.

## Figures and Tables

**Table 1:** Summary of metabolic-based therapeutic drugs to be exploited in pediatric leukemia, including both compounds that directly target metabolic enzymes together with signaling cascade inhibitors that are involved in cell metabolism and its rewiring.

Drug	Target	Condition	Reference
2-deoxy-d-glucose	Hexokinase II	ALL AML	[41] (Leni et al., 2017) [57] (Larrue et al., 2015)
L-Asparaginase	Asparagine availability	ALL AML	[46] (Serravalle et al., 2016) [58] (Bertuccio et al., 2017)
BPTES	Glutaminase	ALL	[59] (Herranz et al., 2015)
Etomoxir	Fatty acid oxidation	ALL	[49] (Hermanova et al., 2016)
IACS 010759	OXPHOS	AML	[60] (Molina et al., 2018)
Metformin	Mitochondrial Complex I	ALL	[45] (Trucco et al., 2018)
Sirolimus Temsirolimus Everolimus	mTOR	ALL AML	[56] (Maude et al., 2012) [61] (Tan et al., 2017)
NVP-BEZ235 BGT226 PI-103 PF-04691502	PI3K/mTOR	AML	[62] (Park et al., 2007) [63] (Kampa-Schittenhelm et al., 2013) [64] (Deng et al., 2017)

## Abbreviations

ALL	Acute Lymphoblastic Leukemia
AML	Acute Myeloid Leukemia
AMPK	AMP-activated protein kinase
ASNase	L-asparaginase
ASNS	Asparagine Synthetase
FAO	Fatty Acid Oxidation
FLT-3	FMS-like Tyrosine Kinase 3
GC	Glucocorticoids
HIF-1	Hypoxia Inducible Factors
HSC	Hematopoietic Stem Cell
LIC	Leukemia-Initiating Cells
LKB1	Liver Kinase B1
LSC	Leukemic Stem Cells
mtDNA	mitochondrial DNA
mTOR	Mammalian Target Of Rapamycin
OXPHOS	Oxidative Phosphorylation
PDK1	Phosphoinositide-dependent Kinase 1
Ph+	Philadelphia Chromosome-positive
PKB	Protein Kinase B
PH	Pleckstrin Homology
RTK	Receptor Tyrosine Kinase
TCA	Tricarboxylic Acid
2-DG	2-deoxy-d-glucose
